# Perinatal exposure to atazanavir-based antiretroviral regimens in a mouse model leads to differential long-term motor and cognitive deficits dependent on the NRTI backbone

**DOI:** 10.3389/fnmol.2024.1376681

**Published:** 2024-04-05

**Authors:** Shreya H. Dhume, Kayode Balogun, Ambalika Sarkar, Sebastian Acosta, Howard T. J. Mount, Lindsay S. Cahill, John G. Sled, Lena Serghides

**Affiliations:** ^1^Toronto General Hospital Research Institute, University Health Network, Toronto, ON, Canada; ^2^Department of Pathology, Montefiore Medical Center and Albert Einstein College of Medicine, Bronx, NY, United States; ^3^Institute of Medical Sciences, University of Toronto, Toronto, ON, Canada; ^4^Tanz Centre for Research in Neurodegenerative Diseases, Department of Psychiatry and Physiology, University of Toronto, Toronto, ON, Canada; ^5^Department of Chemistry, Memorial University of Newfoundland, St. John’s, NL, Canada; ^6^Mouse Imaging Centre, Toronto Centre for Phenogenomics, Toronto, ON, Canada; ^7^Department of Medical Biophysics, University of Toronto, Toronto, ON, Canada; ^8^Women’s College Research Institute, Toronto, ON, Canada; ^9^Department of Immunology, University of Toronto, Toronto, ON, Canada

**Keywords:** HIV antiretrovirals, protease inhibitors, neurodevelopment, cognition, HEU, hyperactivity, memory, MRI

## Abstract

**Background:**

Combination antiretroviral therapy (ART) use in pregnancy has been pivotal in improving maternal health and reducing perinatal HIV transmission. However, children born HIV-exposed uninfected fall behind their unexposed peers in several areas including neurodevelopment. The contribution of *in utero* ART exposure to these deficits is not clear. Here we present our findings of neurocognitive outcomes in adult mice exposed *in utero* to ART.

**Methods:**

Dams were treated with a combination of ritonavir-boosted atazanavir with either abacavir plus lamivudine (ABC/3TC + ATV/r) or tenofovir disoproxil fumarate plus emtricitabine (TDF/FTC + ATV/r), or water as a control, administered daily from day of plug detection to birth. Offspring underwent a battery of behavioral tests that investigated motor performance and cognition starting at 6-weeks of age and ending at 8 months. Changes in brain structure were assessed using magnetic resonance imaging and immunohistochemistry. Expression of genes involved in neural circuitry and synaptic transmission were assessed in the hippocampus, a region strongly associated with memory formation, using qPCR.

**Findings:**

Pups exposed to TDF/FTC + ATV/r showed increased motor activity and exploratory drive, and deficits in hippocampal-dependent working memory and social interaction, while pups exposed to ABC/3TC + ATV/r showed increased grooming, and deficits in working memory and social interaction. Significant volumetric reductions in the brain were seen only in the ABC/3TC + ATV/r group and were associated with reduced neuronal counts in the hippocampus. Altered neurotransmitter receptor mRNA expression as well as changes in expression of the neurotrophic factor BDNF and its receptors were observed in both ART-exposed groups in a sex-dependent manner.

**Interpretation:**

In our model, *in utero* ART exposure had long-term effects on brain development and cognitive and motor outcomes in adulthood. Our data show that neurological outcomes can be influenced by the type of nucleoside reverse transcriptase inhibitor backbone of the regimen and not just the base drug, and display sex differences.

## 1 Introduction

The introduction of antiretroviral (ARV) therapy (ART) for the prevention of perinatal HIV transmission has dramatically reduced the number of children born with HIV worldwide (De Cock et al., 2000; Afran et al., 2014; Toledo et al., 2021; Wedderburn et al., 2022b). Consequently, the population of children who are HIV-exposed but uninfected (CHEU) is on the rise, with approximately 16 million CHEU living across the globe currently (Evans et al., 2016; Slogrove et al., 2020; AIDSinfo | UNAIDS, 2024).

While the benefits of ART outweigh the risks, our knowledge of the safety and toxicity of ART in pregnancy is limited, and there is a paucity of information on the ART effects on fetal development and the health trajectory of these children. CHEU are at higher risk for prematurity and growth restriction, both of which are risk factors for developmental delays. Studies have also reported developmental, cognitive, and behavioral delays in CHEU compared to their unexposed peers (Le Doaré et al., 2012; Sirois et al., 2013; Nozyce et al., 2014; Williams et al., 2016; McHenry et al., 2018; Boivin et al., 2019; Springer et al., 2020; Toledo et al., 2021; Wedderburn et al., 2022b; Yao et al., 2023). These can lead to poorer academic performance and social interactions, and place these children at risk for persistent problems into adulthood (Evans et al., 2016; Black et al., 2017; McHenry et al., 2018; Wedderburn et al., 2019a).

Boosted protease inhibitors administered with a dual nucleoside reverse transcriptase inhibitor (NRTI) backbone are part of recommended regimens for use in pregnancy (Powis et al., 2019; see Table 14 in Department of Health and Human Services, 2024). *In utero* exposure to protease inhibitors such as atazanavir has been associated with delayed emergence of language, hearing, and lower scores on the Bayley Scale III (Rice et al., 2013; Sirois et al., 2013; Himes et al., 2015; Desmonde et al., 2016). Additionally, protease inhibitor-based ART has been associated with higher risk for premature birth particularly with exposure in the first trimester (Powis et al., 2011; Favarato et al., 2018; Shinar et al., 2022), as well as altered *in utero* hormonal environment and placental dysfunction, all of which can influence neurodevelopment (Dunk and Serghides, 2022). Exposure to NRTIs has been associated with an increased risk of mitochondrial dysfunction including in the brain of CHEU (Budd et al., 2018). A recent study assessing neurodevelopmental outcomes in 5-year-old CHEU reported that those exposed *in utero* to ritonavir-boosted atazanavir (ATV/r) with tenofovir/emtricitabine backbone showed a higher risk of single and multiple neurodevelopmental problems (Yao et al., 2023). Although these studies show neurocognitive impairments related to specific antiretroviral drugs, limited studies correlate neurodevelopmental outcomes to specific ART regimens. There is a need to improve our understanding of how perinatal ART exposure affects developmental mechanisms of neurocognition and behavior, to inform selection of ARVs that will optimize maternal and child health.

Investigation of neurocognitive phenotypes in CHEU can be challenging as neurocognitive development can be influenced by multiple factors including environmental and socioeconomic factors. Animal studies enable fundamental and mechanistic studies that are otherwise challenging in human studies. We have previously shown that *in utero* exposure to protease inhibitor-based ART in a mouse model led to somatic growth impairments, olfactory deficits, and delays in primitive reflexes that involve sensory and vestibular motor pathways (Sarkar et al., 2020). As perinatal brain development forms a foundation for long-term behavioral, motor, and cognitive outcomes (Melillo, 2011), this study focused on the long-term effects of perinatal ART exposure on neurocognitive and motor outcomes. We compared motor performance, anxiety-related behaviors, and hippocampal-dependent memory in adult mice perinatally exposed to either water as a control, or ritonavir-boosted atazanavir (ATV/r) administered with either abacavir/lamivudine (ABC/3TC) or tenofovir disoproxil fumarate/emtricitabine (TDF/FTC). Further, we studied the mechanisms underlying the neurocognitive outcomes using magnetic resonance imaging (MRI), tissue immunofluorescence, and gene expression analysis.

## 2 Methods

### 2.1 Ethics statement

Animal experiments conformed to the policies and guidelines of the Canadian Council on Animal Care and were approved by the University Health Network Animal Use Committee.

### 2.2 Breeding

Male and female (8 week-old) C57BL/6 J were purchased from Jackson Laboratory. Animals were housed in a temperature-controlled room at 21 ± 1°C and 55 ± 5% relative humidity under a 12-h light/dark cycle. Food and water were provided *ad libitum*. Female mice were trained on gavage using water for 1 week prior to mating to reduce gavage-related stress during pregnancy. Two female virgin mice were mated with an experienced male. Mating was confirmed by the presence of vaginal plug. Plugged mice were randomly assigned to one of the three treatment arms: ABC/3TC + ATV/r (100/50 mg/kg/day +50/16.6 mg/kg/day), TDF/FTC + ATV/r (50 mg/33.3/kg/day +50/16.6 mg/kg/day), or water as control. Dosing was calculated based on previous pharmacokinetic studies in mice that optimized doses to yield Cmax plasma levels similar to levels reported in humans (Kala et al., 2018; Sarkar et al., 2020; Gilmore et al., 2021; Balogun and Serghides, 2022). Mice received daily treatment by oral gavage in a total volume of 100 μL, starting on day of plug detection until time of delivery of litter. Pups were kept with their mothers until weaning on postnatal day 21. Pups were then sexed and divided into three arms based on their maternal treatment – a total of 55 pups (27 female, 28 male) from 17 litters in the control arm, 48 pups (27 female, 21 males) from 14 litters in the ABC/3TC + ATV/r arm, and 43 pups (21 female, 22 male) from 12 litters from the TDF/FTC + ATV/r arm were included in the behavioral testing.

### 2.3 Behavioral testing

**Figure 1:**
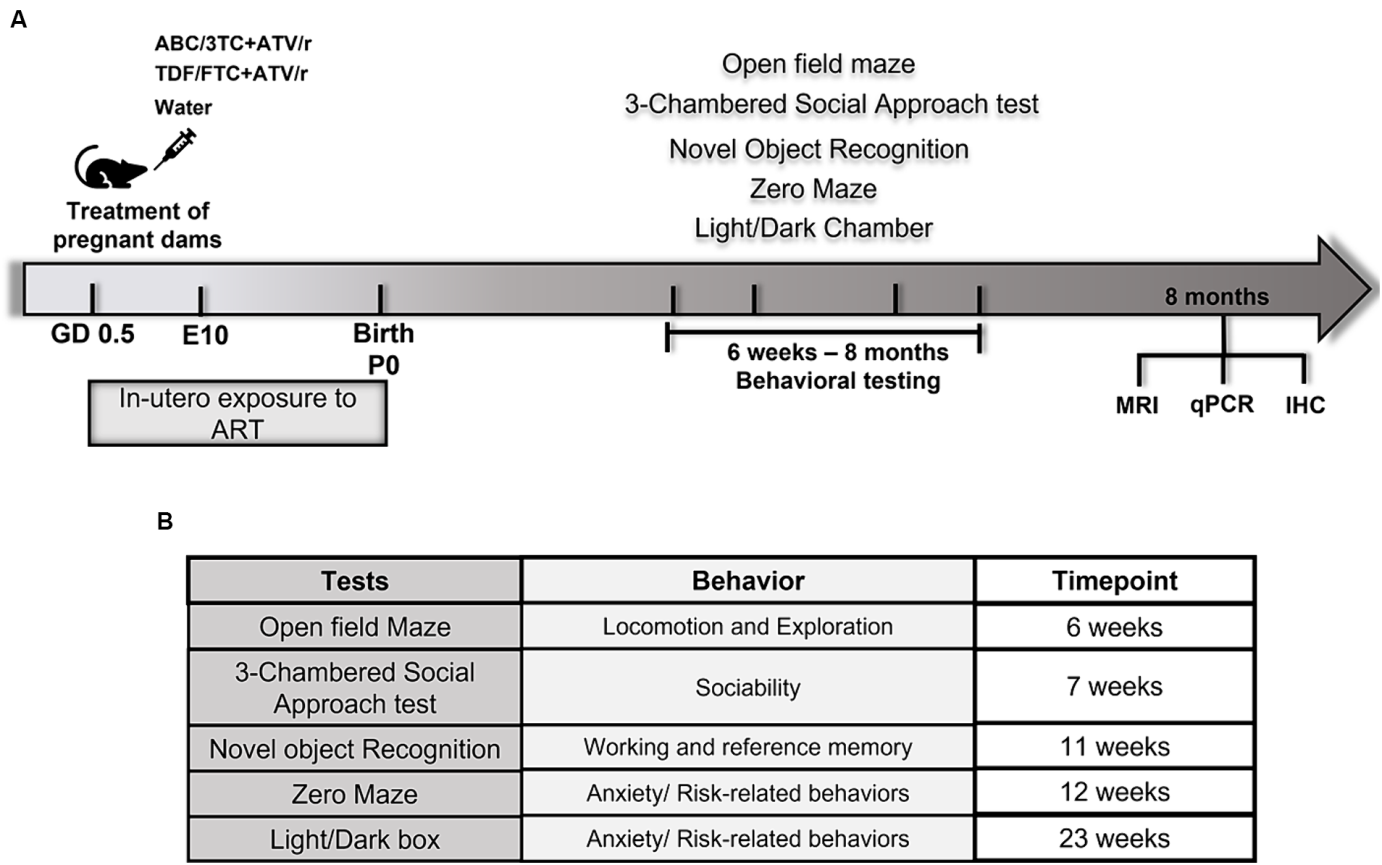
Schematic diagram of experimental design and behavioral tests in mice. **(A)** Pregnant mice were randomly assigned treatment groups of ritonavir-boosted atazanavir combined with either abacavir/lamivudine (ABC/3TC + ATV/r), or tenofovir/emtricitabine (TDF/FTC + ATV/r) and water as control from gestational day 0.5. Behavioral testing was conducted from 6 weeks postnatally followed by brain analyses including quantitative real-time PCR, immunohistochemistry, and magnetic resonance imaging (MRI). **(B)** List of behavioral tests performed on *in utero* exposed mice and the behaviors assessed by the respective tests.

Animals were tested in the following battery of tests in the order presented in Figure 1, beginning at 6 weeks and ending at 8 months of age (N = 21–28 for all treatment groups). All testing took place in an empty behavioral testing room absent of spatial or visual cues. Mice were brought to the testing arena in their home cage and were tested individually. Between tests, the testing cage was thoroughly cleaned with Virox5 (Johnson Diversey, Inc., Sturevant, WI, United States) to eliminate odor cues.

The Open Field Test: The open field test was used to assess exploratory and locomotor behavior, and to assess response to open field stress. Animals were placed in a clean, empty open field (40 cm x 40 cm x 50 cm) made of plywood painted white on the inside floor and walls and black on the outside walls with non-toxic acrylic paint. Animals freely explored the maze for 30 min and their behavior was recorded using ANYMAZE^®^ and ODLog^™^ software. Lighting levels were 330 lux. The following behaviors were coded: immobility; grooming (engaged in self-cleaning behavior), rearing (both front paws raised off of the floor), locomotion (total distance traveled and speed of travel), time spent in the center, and time spent by the wall (thigmotaxis zone).

The Zero Maze Test: Mice were removed from their home cage in the testing room and placed at the entrance of an enclosed region of the maze. Each mouse was allowed to freely explore the maze for a 5-min period after which it was returned immediately to its home cage. Tests were video recorded using ANYMAZE^®^ software. Lighting levels were 330 lux. The total time spent in the enclosed and unenclosed arms were coded and a percentage of time spent in the open arms was calculated.

Light/Dark Box Test: This test assesses unconditioned anxiety responses in mice. The light/dark box has 2 compartments separated by a 7 cm door - a light area (2/3 of the area) and a dark area (1/3 of the area; Bourin and Hascoët, 2003). Each mouse was placed in the light area and allowed to freely explore for 10 min. The time spent in each of the areas was recorded. Tests were video recorded using ANYMAZE^®^ software. Lighting levels in the light box were 580 lux.

The Novel Object Recognition Test: Mice are habituated to the testing arena (clear plastic mouse cages) for 15 min over 7 daily sessions and were considered successfully habituated when they consumed a small piece of breakfast cereal (Fruit Loops) within 2 min of the entrance to the testing arena. On the novel object recognition test day, each animal was exposed for 10 min to a LEGO^®^ construct (single LEGO brick; LEGO Group, Billund, Denmark) and a Hot Wheels^®^ car (model Corvette Z06; Mattel, Inc., El Segundo, CA, United States). The objects were previously determined to be of matched saliency in mice. Objects were fixed to the floor of the mouse cage with magnets. All tests were recorded using ANYMAZE^®^ software. Time spent exploring the objects was recorded using ODLog^™^ software. Exploration was scored when the mouse touched an object with its forepaws or snout, bit, licked, or sniffed the object from a distance of no more than 1.5 cm. Following a 3-h delay, mice were re-exposed for 5 min to one object from the original test pair and to a novel object. The position and shape of the novel object was counterbalanced between treatment groups to control for bias. Lighting levels during all testing were 330 lux.

The 3-Chamber Social Approach Test: The test was done according to the following method (Kaidanovich-Beilin et al., 2011). Lighting levels during testing were 330 lux. Briefly, the sociability test was initiated by habituating the test mouse to an empty 3-room-chamber apparatus for 5 min. An unfamiliar mouse (same sex, background, age, and weight as the test mouse)—stranger 1—was placed in a wire cup in one room of the 3-room chamber. An empty wire cup was placed in the opposite room. The test mouse was released in the middle room and had free access to explore the rooms. The duration and frequency of direct contacts between the test mouse and the empty wire cup or the wire cup housing the unfamiliar mouse were recorded for 10 min. Following the sociability test, the social novelty test was initiated by placing a second unfamiliar mouse—stranger 2—in an identical wire cup in the opposite chamber for 10 min. The duration and frequency of direct contacts between the test mouse and the wire cup housing the familiar mouse (stranger 1) or the wire cup housing the novel mouse (stranger 2) were recorded for 10 min. Normal sociability for a mouse is to prefer to spend time with another mouse rather than the empty cup, and also show preference for social novelty; any deviation from this indicates impaired sociability (Kaidanovich-Beilin et al., 2011). A “sociability index” was calculated as (T_S1_-T_WC_)/(T_S1_ + T_WC_), wherein “T_S1_” represents the duration spent with stranger 1 and “T_WC_” the duration spent exploring empty wire cup. A “social novelty index” was calculated as (T_S1_-T_S2_)/(T_S1_ + T_S2_), wherein “T_S1_” represents the duration spent with stranger 1 and “T_S2_” the duration spent with stranger 2 or novel mouse.

Brain collection: At 8 months of age, following completion of all behavioral testing, mice were sacrificed and brains were collected for use in either magnetic resonance imaging, immunofluorescence imaging, or expression analyses.

### 2.4 Magnetic resonance imaging

Sample preparation: To prepare the brains for neuroanatomical assessment, mice underwent trans-cardiac perfusion (*N* = 14–24 per treatment group; Cahill et al., 2012). Briefly, mice were anesthetized with ketamine/xylazine and perfused through the left ventricle with 30 mL PBS and 2 mM ProHance (Bracco Diagnostics, Inc., NJ, United States), followed by 30 mL of 4% Paraformaldehyde (PFA) and 2 mM ProHance. The brains within the skull were incubated in 4% PFA containing 2 mM ProHance overnight at 4°C and then stored in a solution containing PBS, 2 mM ProHance, and 0.01% sodium azide for at least 28 days before imaging (de Guzman et al., 2016).

Imaging: A multi-channel 7.0 T magnet (Varian Inc. Palo Alto, CA, United States) and a custom-built solenoid array were used to image 16 samples in parallel (Dazai et al., 2011). An anatomical scan was performed using a T2-weighted, 3D fast spin-echo sequence with a cylindrical k-space acquisition and the following parameters: TR = 350 ms, TE = 12 ms, echo train length = 6, four averages, field-of-view 20 mm x 20 mm x 25 mm, matrix size = 504 × 504 × 630, isotropic image resolution = 40 μm (Spencer Noakes et al., 2017). Five control and one ABC/3TC + ATV/r brain had to be excluded from analysis because of damage during preparation for imaging or image misregistration.

Volume analysis: An automated image registration approach implemented in the Pydpiper toolkit (Friedel et al., 2014) and using the Advanced Normalization Tools (ANTs) deformation algorithm (Avants et al., 2008) was used. The 53 MR images were registered together through a process of linear and non-linear steps to create an average image (Nieman et al., 2018). The registration yielded deformation fields for each brain, and the absolute and relative Jacobian determinants provided an estimate of the local volume changes at every voxel for comparison between the groups (Chung et al., 2001). Using the results of the linear alignment, multiple templates of a segmented anatomical atlas with 182 labeled structures (Dorr et al., 2008; Ullmann et al., 2013; Steadman et al., 2014) were created (the MAGeT procedure; Chakravarty et al., 2013). From the final voted segmentation, volume changes were calculated and expressed in absolute volumes (mm^3^). These volumes were then normalized to total brain volume.

### 2.5 Tissue immunofluorescence and image analysis

Immunofluorescence studies were performed on paraffin-embedded fixed brain tissue (*N* = 10 for all treatment groups). Mice were anesthetized and perfused through the left ventricle with 30 mL PBS, followed by 30 mL of 4% PFA. The brains were then extracted, post-fixed in 4% PFA overnight at 4°C and then stored in a solution containing PBS and 0.01% sodium azide. Brains were processed and embedded in paraffin, and then coronally sectioned (6 um thick) and mounted on Superfrost Plus slides (20 slides series with 4 slices per slide). Antigen retrieval was performed by boiling slides in citrate buffer using a microwave (4 min at power 6 followed by a cool down and again at 3 min at power 6). Slides were then incubated in DAKO serum-free blocking solution (Agilent Technologies) for 45 min at room temperature, followed by incubation with primary antibodies suspended in PBS, either NeuN at 1:200 (Abcam; ab177487), or GFAP at 1:1000 (Abcam; ab4674) for 18–24 h at 4°C. Slides were washed in PBS for 9 min, and incubated with appropriate secondary antibodies conjugated to Alexa 488 or Alexa 647 (Abcam), and DAPI (4′,6 diamidino-2-phenylindole) for 1.5 h at room temperature. Prolong Diamond antifade mounting medium was used (ThermoFisher P36961). Slides were scanned with a Zeiss Axioscan 7 microscope slide scanner (ZEISS) at 20x objective, and images were analyzed. For counts, total number of cells were counted in every 6th section to ensure the same neurons were not counted in two sections, and the sections counted spanned the hippocampus (Bregma coordinates −1.35 to −2.6; Malberg et al., 2000) For neuronal counts, NeuN positive cells in the pyramidal layers of CA1 and dentate gyrus were manually counted on ImageJ software. For glial cells, GFAP positive cells co-labeled with DAPI were counted manually using Zen 3.6 Blue software (ZEISS) in the dendritic layers of CA1 and molecular layers of dentate gyrus. All data are reported as the mean ± SEM.

### 2.6 RNA isolation and quantitative PCR

Brains were collected and dissected into specific regions and then snap frozen (N = 20–25 per treatment group). For this study, we isolated total RNA from the hippocampus using the MirVana kit (ThermoFisher AM1560) according to the manufacturer’s instructions. RNA quality and concentration were determined using the Nano-Drop1000 Spectrophotometer (Thermo Fisher Scientific). RNA was treated with DNase I to remove genomic DNA and reverse transcribed into cDNA using iScript^™^ cDNA Synthesis Kit (Biorad 1,725,035) according to the manufacturer’s instructions. Target gene mRNA levels were assessed by quantitative PCR using SsoAdvanced Universal SYBR Green Supermix master mix (Biorad 1,725,270) and the CFX Opus RT-PCR System. The primer sequences for genes are shown in Table 1. Each reaction contained either 50 or 100 ng of cDNA, 1.24 μL of forward and reverse primers, and 3 μL of SYBR and was run in triplicate. The cycling conditions were as follows: 95°C – 3 min, 95°C – 10s, 60°C – 15 s, 72°C – 15 s, repeat 44 times, 65°C – 5 s, 95°C – 0.5°C/cycle. Gene expression was normalized to the geometric mean of 3 housekeeping genes (HPRT, TBP, and PPIA). Relative expression of target genes was obtained using the 2ΔΔCT method (Livak and Schmittgen, 2001).

**Table 1:** Primer sequences.

Gene	Forward primer	Reverse primer
GluA1	CCCTGAGAGGTCCCGTAAAC	GCTCAGAGCACTGGTCTTGT
GluA2	CTACGAGTGGCACACTGAGG	CCCAGAGAGAGATCTTGGCG
GLUN2A	CAAATTCAACCAGAGGGGCG	TGGCAAAGATGTACCCGCTC
GLUN2B	CCTCCTGTGTGAGAGGAAAG	CTCTGTGTGGAGAAGCTGGG
PSD95	AGCCCCAGGATATGTGAACG	TCACCGATGTGTGGGTTGTC
TrkB FL	GACATTCCAAGTTTGGCATGAAAGG	AGAAGATGGAGTGTTACTCCCATT
TrkB TR	GCTGGTGATGTTGCTCCTGC	CCATCCAGTGGGATCTTATGAAAC
BDNF	CTCAGGCAGAATGAGCAATG	AGCCGTCTGTGCTCTTCACT
HPRT	AGCGTCGTGATTAGCGATGA	ACACTTTTTCCAAATCCTCGG
TBP	CCTTGTACCCTTCACCAATGAC	ACAGCCAAGATTCACGGTAGA
PPIA	CCAGGGTGGTGACTTTACAC	ATGCTTGCCATCCAGCCA

### 2.7 Statistical analyses

Statistical analyses were performed using Prism (v8.2) and Stata (v13). All behavioral and expression data analyses were performed stratified by sex, and means with SEM were calculated. Differences in mean with 95% confidence intervals between the control group and the ART arms were calculated using mixed effects linear models with robust standard errors that included treatment as a categorical fixed effect, and litter as a random variable to account for litter effects. Neuronal and glial count data are shown as violin plots with individual data points displayed. Differences were assessed using Student’s t-test. The Pearson r test was used to assess correlations. MRI data analysis was performed using the R statistical software^1^ and the RMINC package.^2^ For the adult brain MR images, sex was included as a fixed effect. Multiple comparisons were controlled for using the false discovery rate (FDR; Genovese NeuroImage 2002, 15:870–878) and statistical significance was defined at an FDR threshold of 1%.

## 3 Results

The experimental design is shown in Figure 1A. Male and female mice exposed *in utero* to either control (water), ABC/3TC + ATV/r, or TDF/FTC + ATV/r were tested with a battery of behavioral tests to assess the motor, cognitive, and social abilities starting at 6 weeks of age (Figure 1B). Means with standard error of mean for each behavioral test by treatment group are shown in Table 2. The impact of *in utero* ART exposure on performance in each test was assess using a mixed effects linear model that included treatment as a categorical fixed effect with control-treatment as the reference, and litter as a random variable to account for litter effects. Analyses were stratified by sex, and data are shown as the difference in mean with 95% confidence interval compared to control (summarized in Supplementary Table S1). The raw data for the behavioral tests are shown in Supplementary Figures S1 and S2. Birth weight (measured at postnatal day 3) was lower in the ABC/3TC + ATV/r compared to the other treatment groups (median (interquartile range) with *p*-value vs. control: for males—control: 1.72 g (1.62, 1.80), ABC/3TC + ATV/r: 1.44 g (1.35, 1.74), *p* = 0.018, TDF/FTC + ATV/r: 1.64 g (1.45, 1.82), *p* = 0.28; for females – control: 1.58 g (1.42, 1.79), ABC/3TC + ATV/r: 1.49 g (1.31, 1.65), *p* = 0.083, TDF/FTC + ATV/r: 1.60 g (1.43, 1.98), *p* = 0.65). To account for possible contributions of lower birth weight on behavioral scores, analyses were repeated adjusting for weight at postnatal day 3. The adjusted difference in mean with 95% confidence interval compared to control are shown in Supplementary Table S2. In general, adjusting for weight did not impact on behavioral differences between treatment groups. At 8 months, mice were sacrificed and assessed for morphological and physiological changes in the brain using MRI, immunohistochemistry, and real-time quantitative PCR.

**Table 2:** Means with standard error of mean (SEM) for behavioral test by treatment group.

	Control Mean ± SEM		ABC/3TC + ATV/r Mean ± SEM		TDF/FTC + ATV/r Mean ± SEM	
	*M*	*F*	*M*	*F*	*M*	*F*
Open field maze						
*Total distance (m)*	37.8 ± 2.1	47.0 ± 2.7	36.3 ± 1.7	39.1 ± 3.0	50.4 ± 3.2*	55.4 ± 3.3*
*Mobile time (s)*	265.3 ± 12.5	326.0 ± 15.4	253.4 ± 11.3	264.9 ± 20.3	330.1 ± 16.7*	370.6 ± 17.3
*Rearing (s)*	79.3 ± 7.5	98.1 ± 10.3	76.6 ± 7.2	78.6 ± 7.0	161.2 ± 11.9***	170.9 ± 9.3***
*Grooming (s)*	50.7 ± 5.6	38.2 ± 3.6	66.5 ± 7.4	70.2 ± 9.2***	36.1 ± 4.2*	26.9 ± 2.8*
*Wall time (s)*	828.5 ± 7.6	835.7 ± 6.1	821.9 ± 54.6	826.0 ± 9.4	835.2 ± 7.3	845.8 ± 5.6
*Center time (s)*	69.9 ± 8.0	62.4 ± 6.1	67.0 ± 10.3	68.9 ± 6.8	63.6 ± 6.7	60.7 ± 5.5
Light/dark box						
*Time in light box (s)*	188.3 ± 10.8	169.8 ± 6.9	190.0 ± 13.1	167. 6 ± 9.8	209.8 ± 7.7	202.4 ± 7.1**
Zero maze						
*Time in open area (s)*	34.5 ± 3.6	42.7 ± 4.2	47.4 ± 4.3*	51.7 ± 3.5	55.6 ± 4.8***	57.0 ± 4.2
Novel object recognition						
*Exploration time (s)*	113.8 ± 9.2	139 ± 13.8	121.1 ± 7.5	111.3 ± 7.9	165.9 ± 8.3**	219.5 ± 8.3***
*Time with novel object (s)*	45.0 ± 3.7	44.7 ± 3.6	29.4 ± 3.0***	30.0 ± 3.0	42.8 ± 1.9	56.0 ± 4.0
*Memory Index*	0.22 ± 0.03	0.19 ± 0.03	0.036 ± 0.06*	−0.0003 ± 0.04***	0.11 ± 0.03**	0.089 ± 0.03**
Social approach						
*Sociability Index*	0.27 ± 0.05	0.23 ± 0.03	0.33 ± 0.06	0.25 ± 0.04	0.18 ± 0.05	0.14 ± 0.04**
*Social Novelty Index*	0.43 ± 0.04	0.42 ± 0.05	0.48 ± 0.07	0.25 ± 0.05**	0.47 ± 0.05	0.33 ± 0.05

Statistical comparisons stratified by sex using mixed effects linear models with robust standard errors that included treatment as a categorical fixed effect, and litter as a random variable to account for litter effects. All comparison vs. control. *p < 0.05, **p < 0.01, ***p < 0.001.

### 3.1 *In utero* exposure to TDF/FTC + ATV/r is associated with increased locomotor activity and exploratory behavior, and reduced anxiety

**Figure 2:**
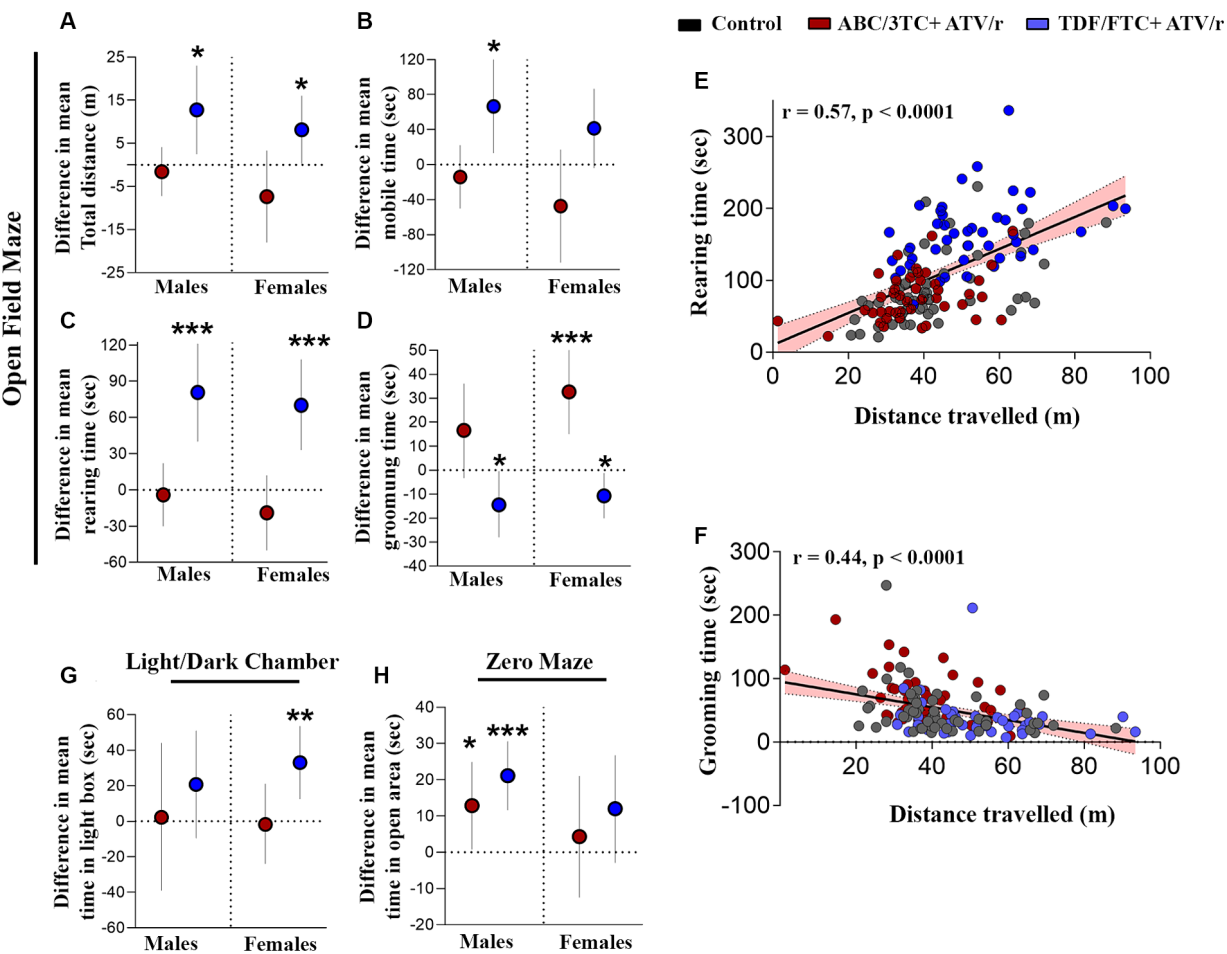
*In utero* exposure to TDF/FTC + ATV/r is associated with increased locomotor activity and exploratory behavior. Difference in mean performance from control (indicated as the dotted line) with 95% confidence intervals are shown for male and female mice exposed to ABC/3TC + ATV/r (red dots) or TDF/FTC + ATV/r (blue dots) for the open field test **(A–D)**, light/dark chamber test **(G)** and zero maze test **(H)**. For the open field test we show difference in mean **(A)** distance traveled, **(B)** time mobile, **(C)** rearing time, and **(D)** grooming time. We also show correlation between rearing and distance traveled **(E)** and between grooming time and distance traveled **(F)**. For the light/dark chamber test we show difference in mean time spent in the light box **(G)**. For the zero maze test we show difference in mean time spent in the open area **(H)**. Mixed effects linear models with robust standard errors were fit to estimate the difference in mean performance, 95% confidence intervals, and *p*-values between ART-exposed mice and controls for each test metric. Litter was included as a random variable to account for litter variability. Correlations by Pearson r. For males: *n* = 21 from 6 litters for ABC/3TC + ATV/r, *n* = 22 from 6 litters for TDF/FTC + ATV/r, and *n* = 28 from 9 litters for control. For females: *n* = 27 from 8 litters for ABC/3TC + ATV/r, *n* = 21 from 6 litters for TDF/FTC + ATV/r, *n* = 27 from 9 litters for control. *p < 0.05, ***p < 0.001. ABC, abacavir; 3TC, lamivudine; TDF, tenofovir; FTC, emtricitabine; ATV/r. ritonavir-boosted atazanavir.

Some studies suggest altered motor skills and increased levels of anxiety in CHEU (Chase et al., 2000; Wedderburn et al., 2019b, 2022a,b; Yao et al., 2023). We first assessed locomotor activity, anxiety, and exploration using the open-field test. Both male and female TDF/FTC + ATV/r-exposed mice showed increased distance traveled (Figure 2A, difference in mean from control for male +12.8 m *p* = 0.01, for female +8.1 m, *p* = 0.04) compared to control and ABC/3TC + ATV/r-exposed mice. Both male and female TDF/FTC + ATV/r exposed mice also showed increased mobile time compared to control and ABC/3TC + ATV/r, although this only reached significance for the males (Figure 2B, difference in mean from control for male TDF/FTC + ATV/r + 66.5 s, *p* = 0.01, for female +41.4 s, *p* = 0.07). When we adjusted for weight at postnatal day 3, the increase in mobile time seen in the TDF/FTC + ATV/r-exposed females did reach significance (Supplementary Table S2). No difference was observed in distance traveled between ABC/3TC + ATV/r-exposed mice and controls for either sex, although female ABC/3TC + ATV/r-exposed mice had a lower mobile time compared to controls that did not reach significance (Figure 2A). Time spent in the center and time spent by the wall (thigmotaxis zone) were similar between groups (Table 2; Supplementary Table S1). These data suggest an increased locomotor activity and exploratory drive with TDF/FTC + ATV/r, but not with ABC/3TC + ATV/r exposure.

We also assessed rearing time in the open field test, another indicator of locomotor activity, exploration, and anxiety. Increased rearing was observed in both male and female TDF/FTC + ATV/r-exposed mice compared to the other groups, again suggesting increased activity and reduced anxiety (Figure 2C, difference in mean from control for male TDF/FTC + ATV/r + 80.6 s, *p* < 0.001, for female +70.2 s, *p* < 0.001). Rearing time did not differ significantly between the ABC/3TC + ATV/r-exposed mice and controls.

Next, we assessed grooming in the open field. Grooming in rodents is a natural response to displacement which is often exhibited upon exposure to a novel environment. In a stressful environment with a high anxiety level, rodents tend to groom longer than usual (Estanislau, 2012; Kalueff et al., 2016; Liu et al., 2021). Both male and female ABC/3TC + ATV/r-exposed mice spent more time grooming compared to controls and TDF/FTC + ATV/r-exposed mice, although this only reached significance for the female mice (Figure 2D, difference in mean from control for male ABC/3TC + ATV/r + 16.6 s, *p* = 0.1, for female +32.6 s, *p* < 0.001), suggesting greater anxiety with ABC/3TC + ATV/r exposure. Supporting a phenotype of reduced anxiety, TDF/FTC + ATV/r-exposed mice had the lowest grooming times (Figure 2D, difference in mean from control for male TDF/FTC + ATV/r − 14.5 s, *p* = 0.04, for female −10.7 s, *p* = 0.02). The significant difference in the TDF/FTC + ATV/−exposed males was lost when we adjusted for weight at postnatal day 3 (Supplementary Table S2). As expected, we observed a strong correlation between rearing time and distance traveled (*r* = 0.57, *p* < 0.0001; Figure 2E). In support of increased grooming being an anxiety-related behavior, we observed a negative correlation between grooming time and distance traveled (*r* = −0.44, *p* < 0.0001; Figure 2F).

Next, we used the zero maze and light/dark box, two tests of exploratory and avoidance-related behaviors that are more sensitive to anxiety-related behavior (Hånell and Marklund, 2014; Thompson et al., 2018; La-Vu et al., 2020). Mice exposed to TDF/FTC + ATV/r spent more time in the light chamber of the light/dark chamber test, although this reached significance compared to controls only in the female mice (Figure 2G, difference in mean from control for male TDF/FTC + ATV/r + 20.8 s, *p* = 0.18, for female +33.0 s, *p* = 0.002). As predicted, male and female mice exposed to TDF/FTC + ATV/r spent more time in the open area of the zero maze compared to controls although this only reached significance for male mice (Figure 2H, difference in mean from control for male TDF/FTC + ATV/r + 21.1 s, *p* < 0.001, for female +11.9 s, *p* = 0.11). Male mice exposed to ABC/3TC + ATV/r also spent significantly more time in the open area compared to controls (mean difference from control for male ABC/3TC + ATV/r + 12.9 s, *p* = 0.036), although significance was lost when we adjusted for weight at postnatal day 3 (Supplementary Table S2).

Collectively our data suggest increased mobility and exploratory behavior, as well as indications of reduced anxiety or increased risk-taking behavior, in mice exposed to TDF/FTC + ATV/r.

### 3.2 Working memory and sociability is impaired in mice exposed *in utero* to ARV regimens

**Figure 3:**
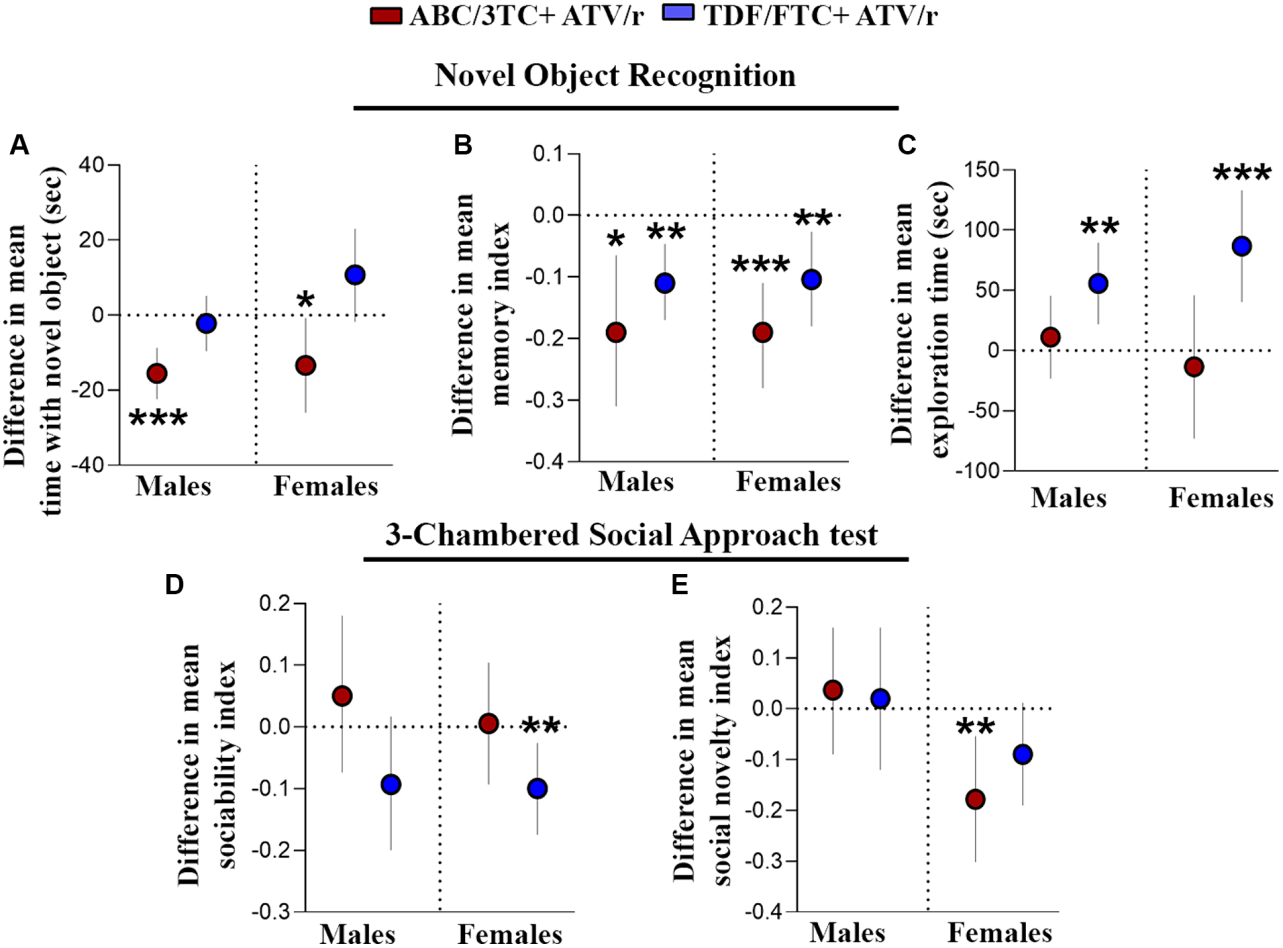
Working memory and sociability is impaired in mice exposed *in utero* to ART. Difference in mean performance from control (indicated as the dotted line) with 95% confidence intervals are shown for male and female mice exposed to ABC/3TC + ATV/r (red dots) or TDF/FTC + ATV/r (blue dots) for the novel object recognition test **(A–C)** and 3-chamber social test **(D,E)**. For the novel object recognition test we show difference in mean **(A)** time with the novel object, **(B)** memory index, and **(C)** exploration time. For the 3-chamber social approach test we show difference in mean **(D)** sociability index and **(E)** social novelty index. Mixed effects linear models with robust standard errors were fit to estimate the difference in mean performance, 95% confidence intervals, and p-values between ART-exposed mice and controls for each test metric. Litter was included as a random variable to account for litter variability. For males: *n* = 21 from 6 litters for ABC/3TC + ATV/r, *n* = 22 from 6 litters for TDF/FTC + ATV/r, and *n* = 28 from 9 litters for control. For females: *n* = 27 from 8 litters for ABC/3TC + ATV/r, *n* = 21 from 6 litters for TDF/FTC + ATV/r, *n* = 27 from 9 litters for control. *p < 0.05, **p < 0.01, ***p < 0.001. ABC, abacavir; 3TC, lamivudine; TDF, tenofovir; FTC, emtricitabine; ATV/r. ritonavir-boosted atazanavir.

Deficits in cognitive abilities have been reported in CHEU as compared to unexposed children, including learning deficits and mild cognitive impairment (Wedderburn et al., 2019a, 2022b; Yao et al., 2023). We first assessed hippocampal-dependent working memory, requiring neural circuits associated with the prefrontal cortex and medial temporal lobe (Frank et al., 2004; Chao et al., 2022; Voitov and Mrsic-Flogel, 2022). The novel object recognition test is a test of non-spatial, episodic memory that is independent of neuromotor deficits and emotional cues. A “memory index” was calculated as (T_N_-T_F_)/(T_N_ + T_F_), wherein “T_N_” represents time exploring a novel object and “T_F_” the duration of familiar object exploration. A positive memory index indicates preference for the novel object, which is normal behavior for mice. As expected, the control mice spent statistically significantly more time exploring the novel object rather than the familiar object, with a mean memory index with SEM of 0.22 ± 0.03 for males and 0.19 ± 0.03 for females (Table 2). The time spent exploring the novel object, as well as memory index, were lower in ABC/3TC + ATV/r treatment group compared to controls for both males and females (Figures 3A,B), with the memory index being close to zero indicating failure to differentiate between the novel and familiar object (memory index with SEM for ABC/3TC + ATV/r: 0.036 ± 0.06 for male, −0.0003 ± 0.04 for female; Table 2). While time spent exploring the novel object was similar between the TDF/FTC + ATV/r group and control, total exploration time was significantly higher in mice exposed to TDF/FTC + ATV/r, which supports the open field results of hyperactivity (Figure 3C, difference in mean from control for male TDF/FTC + ATV/r + 55.7 s, *p* = 0.001, for female +86.7 s, *p* < 0.001). This also resulted in a lower memory index for both male and female mice exposed to TDF/FTC + ATV/r as compared to controls (Figure 3B), with the memory index being 0.11 ± 0.03 for males and 0.089 ± 0.03 for female mice (Table 2). These findings suggest an impairment in working/reference memory encoding in mice exposed to both regimens *in utero*.

We next investigated social memory in our mouse model using 3-chambered social approach test. Mice exposed to TDF/FTC + ATV/r showed reduced performance (lower sociability index) in the first task of the test, although this only reached significance for the female mice (Figure 3D, difference in mean from control for male TDF/FTC + ATV/r − 0.093, *p* = 0.09, for female −0.10, *p* = 0.008). ABC/3TC + ATV/r exposed females showed reduced social novelty (lower social novelty index) in the second task (Figure 3E, difference in mean from control for male ABC/3TC + ATV/r − 0.037, *p* = 0.56, for female −0.18, *p* = 0.005). Females exposed to TDF/FTC + ATV/r also had a lower social novelty index compared to controls although this did not reach significance (*p* = 0.08). Our data suggest that female mice may be more susceptible to sociability deficits due to ART exposure and that NRTI backbone can also influence responses.

### 3.3 *In utero* exposure to ABC/3TC + ATV/r, but not TDF/FTC + ATV/r, leads to brain volumetric reductions that correlate with memory deficits

**Figure 4:**
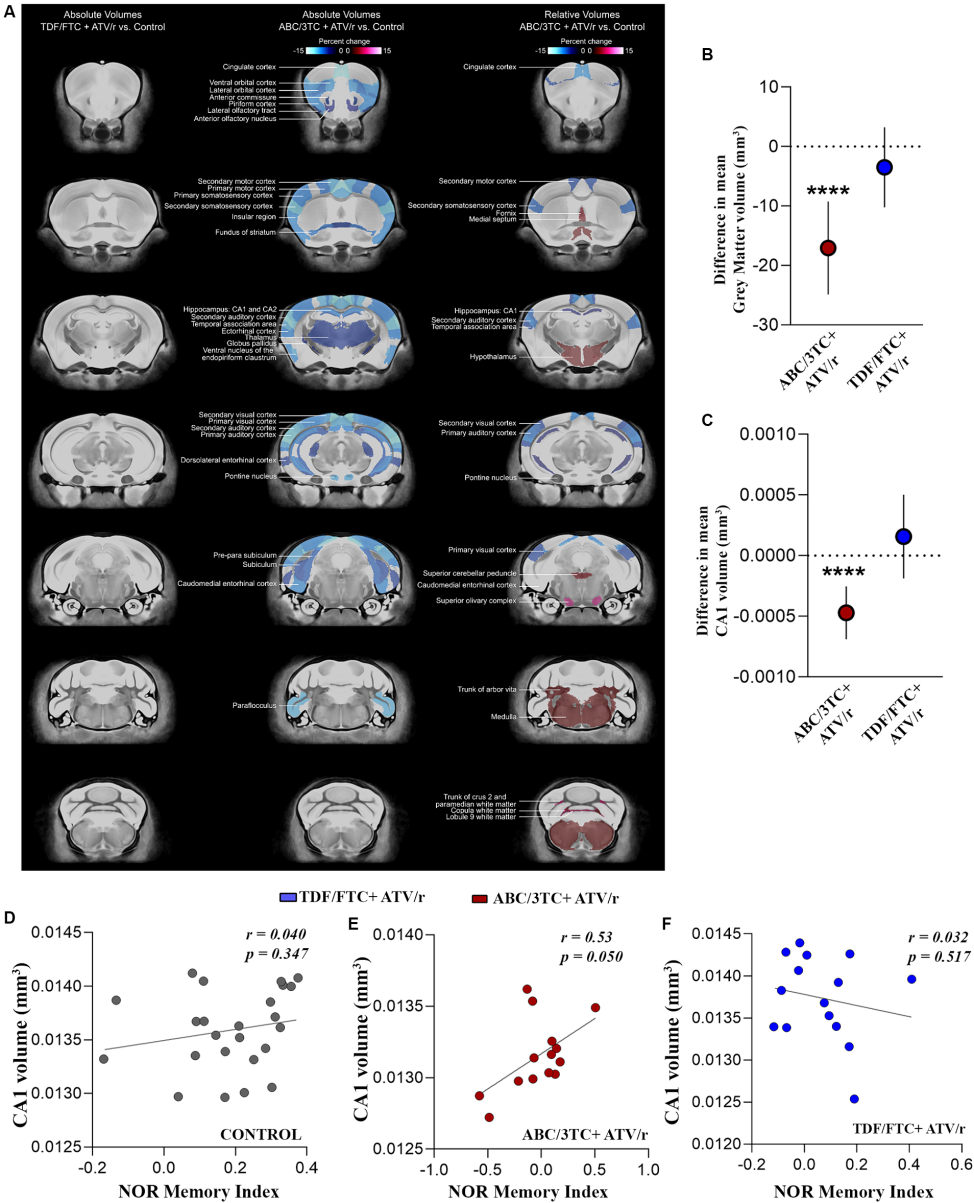
*In utero* exposure to ABC/3TC + ATV/r is associated with volumetric changes in the brain. **(A)** Magnetic resonance imaging of absolute and relative volumes in ABC/3TC + ATV/r and TDF/FTC + ATV/r exposed mice compared to controls (blue regions show decreased volume while red regions show increased volumes). Difference in mean relative volume compared to control for gray matter **(B)** and CA1 **(C)** for ABC/3TC + ATV/r (red dots) or TDF/FTC + ATV/r (blue dots) exposed mice. **(D–F)** Correlation between CA1 relative volume and memory index (novel object recognition test) in controls **(D)**, ABC/3TC + ATV/r exposed mice **(E)**, and TDF/FTC + ATV/r exposed mice **(F)**. Mixed effects linear models with robust standard errors were fit to estimate the difference in mean volume, 95% confidence intervals, and p-values between ART-exposed mice and controls. Litter was included as a random variable to account for litter variability. Correlations by Pearson r. For control, *n* = 24 (11 male, 13 females) from 8 litters; for ABC/3TC + ATV/r, *n* = 14 (7 male, 7 female) from 6 litters; for TDF/FTC + ATV/r, *n* = 15 (8 male, 7 female) from 5 litters. ***p < 0.001. ABC, abacavir; 3TC, lamivudine; TDF, tenofovir; FTC, emtricitabine; ATV/r. ritonavir-boosted atazanavir.

With deficits observed in learning and memory functions of the hippocampus, we next investigated the effect of *in utero* ART exposure on the macroscopic morphology of the brain using magnetic resonance imaging (MRI). At a false discovery rate of 1%, we observed statistically significant reductions in total brain volume in the ABC/3TC + ATV/r group compared to controls. The decrease in total brain volume was not uniform across the brain, with significant brain volume decreases observed primarily in the cortex and thalamus (Figure 4A, center panel). When normalized to account for total brain volume, we also observed significant relative volume differences in brain regions of ABC/3TC + ATV/r exposed mice as compared to controls (Figure 4A, right panel). Of interest areas associated with memory, including the hippocampus, were significantly smaller in the ABC/3TC + ATV/r exposed mice vs. control. No differences were observed in TDF/FTC + ATV/r treatment group compared to controls (Figure 4A, left panel).

When further dissected into regions, ABC/3TC + ATV/r exposed mice showed significantly lower gray matter volume as compared to controls and TDF/FTC + ATV/r exposed mice (Figure 4B, difference in mean from control -17 mm^3^, *p* < 0.0001). The volume of the CA1 region of the hippocampus was also lower in ABC/3TC + ATV/r exposed mice as compared to controls (Figure 4C, difference in mean from control −0.0004mm^3^, *p* < 0.0001). Since there were no significant sex-by-group interactions, for these analyses we combined males and females. We observed a strong positive correlation between CA1 volume and memory index in ABC/3TC + ATV/r exposed mice, but not in the control or TDF/FTC + ATV/r exposed mice (Figures 4D–F).

**Figure 5:**
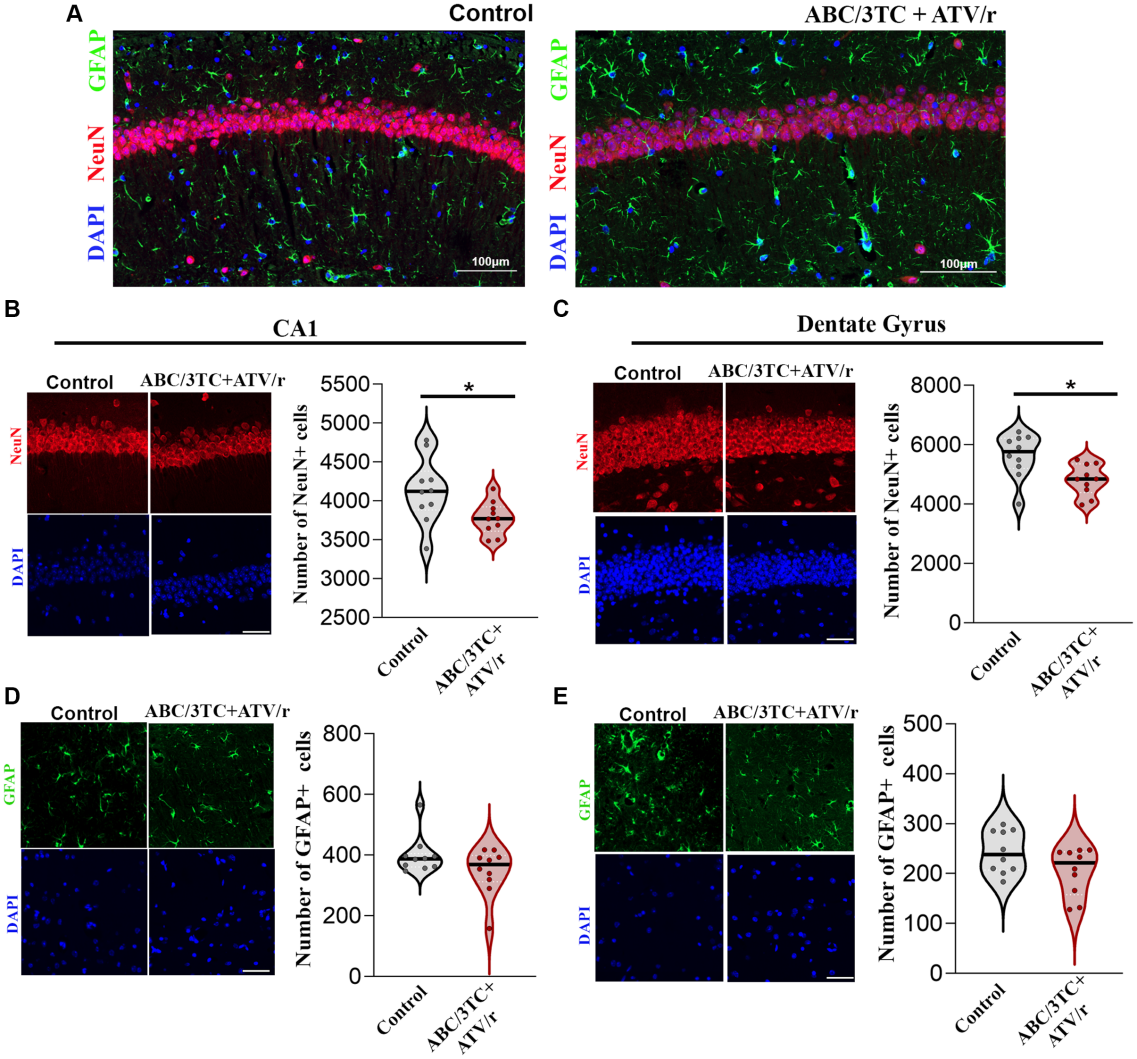
Neuronal, but not glial, populations in CA1 and dentate gyrus of the hippocampus are reduced in ABC/3TC + ATV/r mice. **(A–E)** Representative immunofluorescence images of CA1 region of ABC/3TC + ATV/r and control mice stained with NeuN (red), GFAP (green) and DAPI (blue). Number of neurons (NeuN+ cells) in CA1 **(B)** and dentate gyrus **(C)** of the hippocampus. Number of glial cells (GFAP+) in CA1 **(D)** and dentate gyrus **(E)** of the hippocampus. Data are shown as violin plots with dots indicating counts for each mouse and line indicating mean. Statistical comparisons using Student’s t-test; *p < 0.05. *N* = 10 (5 male, 5 female) per treatment group. Scale bar is 50 μm. ABC, abacavir; 3TC, lamivudine; TDF, tenofovir; FTC, emtricitabine; ATV/r. ritonavir-boosted atazanavir.

Supporting the MRI findings of smaller hippocampal volume, immunostaining of paraffin-embedded fixed sections of the brain with the neuronal marker NeuN, showed a decreased count of pyramidal neurons in the CA1 and dentate gyrus granule cells of ABC/3TC + ATV/r exposed mice compared to controls (Figures 5A–C). We also investigated changes in the number of glial cells using glial fibrillary acidic protein (GFAP) as a marker, but observed no differences between treatment groups (Figures 5D,E). These results support an association between brain volumetric reductions, reduced neuronal counts, and memory deficit in mice exposed to ABC/3TC + ATV/r. In contrast TDF/FTC + ATV/r exposure was not associated with macroscopic brain volumetric differences.

### 3.4 Altered gene expression in the hippocampus of mice exposed i*n utero* to ABC/3TC + ATV/r and TDF + FTC/ATV/r

**Figure 6:**
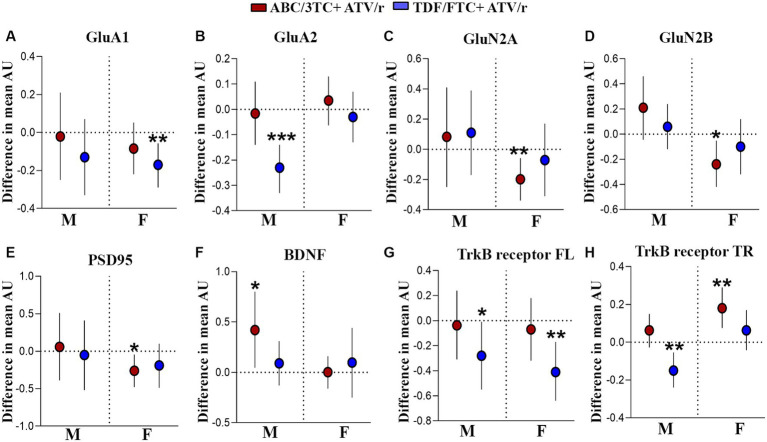
Altered gene expression in the hippocampus of mice exposed to ABC/3TC + ATV/r and TDF + FTC/ATV/r *in utero*. Difference in mean hippocampal gene expression (arbitrary units) from control (indicated as the dotted line) with 95% confidence intervals are shown for male and female mice exposed to ABC/3TC + ATV/r (red dots) or TDF/FTC + ATV/r (blue dots) for GluA1 **(A)**, GluA2 **(B)**, GluN2A **(C)**, GluN2B **(D)**, PSD95 **(E)**, BDNF **(F)**, TrkB-full length receptor **(G)**, TrkB-truncated receptor **(H)**. Mixed effects linear models with robust standard errors were fit to estimate the difference in mean expression, 95% confidence intervals, and *p*-values between ART-exposed mice and controls. Litter was included as a random variable to account for litter variability. For control, *n* = 20 (11 male, 9 female) from 11 litters; for ABC/3TC + ATV/r, *n* = 25 (11 male, 14 female) from 12 litters; for TDF/FTC + ATV/r, n = 25 (12 male, 13 female) from 11 litters. *p < 0.05, **p < 0.01, ***p < 0.001. ABC, abacavir; 3TC, lamivudine; TDF, tenofovir; FTC, emtricitabine; ATV/r. ritonavir-boosted atazanavir.

We next investigated if there are molecular changes in the brain that could underly the observed behavior phenotypes in our model. Experience-dependent modification of neural circuits occurs through changes in synaptic transmission and synaptic plasticity (Malenka, 2003; Nicoll, 2003; Citri and Malenka, 2008; Südhof and Malenka, 2008). Two glutamate neurotransmitter receptors that drive synaptic plasticity in the hippocampus of the mammalian brain are amino-3-hydroxy-5-methyl-4-isoxazolepropionic acid receptor (AMPAR) and N-methyl-D-aspartate receptor (NMDAR; Lüscher and Malenka, 2012; Penn et al., 2017). Further, postsynaptic density protein-95 (PSD95) is a major regulator of synaptic maturation by interacting, stabilizing, and trafficking NMDARs and AMPARs to the postsynaptic membrane in the neurons (Kornau et al., 1995; Schnell et al., 2002). *In utero* exposure to ART led to deficits in hippocampal-dependent working memory in our mouse model and increased exploration. Thus, we investigated changes in gene expression of major neurotransmitter receptors affecting memory encoding in the hippocampus. We identified lower mRNA expression of AMPAR subunits GluA1 and GluA2 (Figures 6A,B; Supplementary Table S1) in TDF/FTC + ATV/r exposed females and males, respectively. In contrast, we observed lower mRNA expression of the NMDAR subunits GluN2A and GluN2B in ABC/3TC + ATV/r exposed females (Figures 6C,D; Supplementary Table S1). Lower mRNA expression levels of the postsynaptic scaffolding protein PSD95 were also observed in ABC/3TC + ATV/r exposed females compared to controls (Figure 6E; Supplementary Table S1).

Next, we assessed potential neurotrophic factors that are associated with modulation of neurotransmitter receptors. Several studies have reported the role of neurotrophic signaling of brain-derived neurotrophic factor (BDNF) and its downstream modulatory effect on the expression of AMPARs (Martinez-Turrillas et al., 2002; Martínez-Turrillas et al., 2005; Jourdi et al., 2009; Autry and Monteggia, 2012; Keifer, 2022; Gliwińska et al., 2023). Not only does BDNF signaling promote the formation of new synapses and exert an effect on the pre-synapse, but it also plays a role in promoting synaptic plasticity in the post-synapse (Jourdi et al., 2009; Autry and Monteggia, 2012; Diering and Huganir, 2018; Park, 2018). Given the role of BDNF signaling in the brain, we assessed the expression of BDNF and its receptors, tyrosine kinase B (TrkB) full-length and TrkB truncated in the hippocampus. We observed statistically significantly higher mRNA levels of BDNF in ABC/3TC + ATV/r exposed males (Figure 6F; Supplementary Table S1). Further, we observed lower mRNA levels of the BDNF receptors TrkB full-length (both males and females) and TrkB truncated (males only) in TDF/FTC + ATV/r exposed mice compared to controls (Figures 6G,H; Supplementary Table S1). In contrast, we found upregulation of the TrkB truncated in ABC/3TC + ATV/r exposed females (Figure 6H; Supplementary Table S1). Thus, our findings suggest that *in utero* exposure to ART regimes can lead to an alteration in the receptors associated with synaptic plasticity and neuromodulator proteins in the hippocampal region of the brain.

## 4 Discussion

Using a pregnant mouse model, we demonstrated that *in utero* exposure to ART regimens that contained atazanavir (protease inhibitor) with two different NRTI backbones are associated with long-term adverse neurodevelopmental outcomes in progeny adult mice. Our investigation led us to the following conclusions: *In utero* exposure to ART that contained the NRTI backbone TDF/FTC was associated with increased risk-taking and exploratory behavior in the open field maze, zero maze and light/dark box. Exposure to ART that contained ABC/3TC or TDF/FTC led to impairments in working memory. Volumetric reductions and morphological changes in gray matter were observed in mice that were exposed to ART that contained ABC/3TC, including the hippocampus. Gene expression analysis of the hippocampus showed changes in neurotransmitter receptor composition and alterations in proteins that are involved in transmission and neurotrophic signaling in the brain of mice exposed to both ART regimens.

Differences in the NRTI backbone are often not considered in neurodevelopmental studies, but our findings suggest that the NRTI backbone can influence neurocognitive outcomes. We observed a stark contrast between the two different NRTI backbones tested and the neurodevelopmental outcomes observed. Mice exposed to TDF/FTC showed enhanced exploratory drive and decreased anxiety as observed by increased total distance traveled, mobile time, and rearing in the open field test, increased time spent in the open area of the zero maze test, increased duration in the light chamber of light/dark box test, and increased exploration in the novel object recognition test. Mice exposed to ABC/3TC showed increased grooming reminiscent of anxiety-like behavior, although we did not observe any increased anxious behavior in other tests. Both regimens were associated with statistically significant deficits in working memory. Sociability/social novelty deficits were observed with both regimens but primarily in female mice. Additionally, ABC/3TC-exposed mice had statistically significant brain volumetric and morphological deficits when compared to control mice, while mice exposed to TDF/FTC did not. Our data support the inclusion of not only class-based but also individual drug-based investigations in future studies, and additional considerations on the NRTIs used within each ART regimen.

Adverse motor outcomes, gross motor delays, as well as hyperkinetic syndrome have been observed in CHEU (Sanmaneechai et al., 2005; Van Rie et al., 2008; Budd et al., 2018; Le Roux et al., 2018; Piske et al., 2018; Wedderburn et al., 2019b), although associations with specific ARV exposures is difficult to surmise given the multiple exposures and confounding factors that can influence outcomes and are difficult to control. The combined phenotype of increased exploration and risk-taking behavior, and sociability deficits seen in mice exposed to TDF/FTC + ATV/r in our study is reminiscent of attention-deficit-hyperactive disorder (ADHD). Higher rates of ADHD have been reported in CHEU that correlated with deficits in executive function (Burkey et al., 2015).

*In utero* exposure to either NRTI backbone in the presence of ATV/r resulted in deficits of hippocampal-dependent memories, with both regimens being associated with lower memory index in the novel object recognition test as compared to controls. Our results support previous studies investigating the impact of NRTI exposure on cognitive and learning tasks in rodents (McHenry et al., 2019). Deficits in spatial and working memory tests using Morris water maze were observed in rats treated with ART containing TDF/3TC and the non-nucleoside reverse transcriptase inhibitor (NNRTI), efavirenz (Akang, 2019). Similar to our findings, mice perinatally exposed to either monotherapy or dual therapy with zidovudine and 3TC showed a trend of delayed acquisition and retention of trials in passive avoidance tests (Calamandrei et al., 1999a,b; Venerosi et al., 2001). However, no negative impact on working or reference memory was observed in CD-1 mice perinatally exposed to 3TC monotherapy (Calamandrei et al., 2000; McHenry et al., 2019). Deficits in learning and acquisition have also been reported in CHEU exposed to protease inhibitor and NRTI-containing regimens, including working memory deficits, lower verbal retention scores, and executive memory function deficits, and may contribute to the higher frequency of reading and math impairments observed in school age CHEU (Chase et al., 2000; Nozyce et al., 2014; Caniglia et al., 2016; Sirois et al., 2016; Milligan and Cockcroft, 2017; Rice et al., 2018; Cockcroft and Milligan, 2019; Young et al., 2021; Wedderburn et al., 2022b).

Our findings also showed a small but significant deficit in sociability tasks with both ART regimens, with more pronounced effects seen in female mice. Difficulty in social settings and social neurodevelopment have been strongly reported in CHEU, including lower sociability scores compared to HIV unexposed children (Rencken et al., 2022), and poorer academic performances due to communication issues and social interaction with peers in school settings (Wedderburn et al., 2019a,b; Madlala et al., 2020; Rencken et al., 2022; Mensi et al., 2023). This is the first study that reports social deficits in mice perinatally exposed to clinically relevant doses of combination ART, although sex-specific changes in social behavior were reported in CD-1 mice perinatally exposed to AZT (Venerosi et al., 2003). Our data suggest that ARV exposure may contribute in part to sociability deficits, and support the inclusion of sex as a variable in sociability studies of CHEU.

To begin to understand the mechanisms that could underly the deficits observed in our study we examined brain volumetric reductions, as well as hippocampal gene expression profiles of neurotransmitter receptors and neurotrophic factors that play a central role in neurophysiology of the observed deficits (Wang, 1999; Newcomer and Krystal, 2001; Lee and Kesner, 2002; Sanderson et al., 2008; Baddeley et al., 2011; Sanderson and Bannerman, 2012; Abad-Perez et al., 2023). Mice exposed to ABC/3TC + ATV/r, but not those exposed to TDF/FTC + ATV/r, had significant reduced volume in several brain regions compared to controls, primarily in the gray matter within the striatum, medulla, midbrain, temporal lobe, entorhinal cortex, lateral septum, and hippocampus. Similar changes in brain volume have been observed in CHEU, including reduction in gray matter, as compared to unexposed children that correlated to poorer maternal immune status (Wedderburn et al., 2022a). Our data suggest that, in addition to HIV and maternal immune status, ARV exposure may also play a role in brain volumetric changes in CHEU, and that gray matter may be more susceptible to NRTI exposure. Our observations that neuroanatomical volumetric alterations were observed in only the ABC/3TC-containing ATV/r-based regimen, and the absence of significant changes in the TDF/FTC + ATV/r-exposed mice, suggest an NRTI effect on brain volume, as well as differential responses to different NRTIs. It is possible that poor penetration of protease inhibitors across the placenta and blood–brain barriers may limit their effects on fetal brain development. In contrast NRTIs can pass freely through such barriers. In fact, ABC and 3TC, but not ATV/r, were detectable in fetal brains of mice exposed *in utero* to ABC/3TC-ATV/r (Gilmore et al., 2021).

The reductions in hippocampal volume we observed in ABC/3TC-exposed mice may be attributable to neuronal loss. We observed lower neuronal numbers, but not glial cells, in the hippocampus of ABC/3TC-exposed mice. The hippocampus is one of the brain regions affected in the context of fetal growth restriction or prenatal compromise, leading to neuronal loss and reduced volumes, as well as abnormal neuronal function in the spared neurons (Mallard et al., 2000; Dieni and Rees, 2003; Illa et al., 2013; Basilious et al., 2015). Increased neurodegeneration, including shrinkage of cytoplasm and pyknotic nuclei in CA1 and dentate gyrus, has been reported in Wistar rats exposed to TDF/3TC and efavirenz (Akang, 2019). Multiple *in vitro* studies report oxidative and mitochondrial stress, neurotoxicity and astrocyte senescence in cultured neurons and astrocytes exposed to monotherapy or combined antiretroviral therapy (Ellis et al., 2007; Liner et al., 2010; Robertson et al., 2012; Akay et al., 2014; Blas-García et al., 2014; Shah et al., 2016; Cohen et al., 2017; Nooka and Ghorpade, 2017; Sanchez and Kaul, 2017; De Benedetto et al., 2020; Bertrand et al., 2021; Lanman et al., 2021; Foster et al., 2022; Rudd and Toborek, 2022). While these studies investigated the direct neurotoxic effects of ARVs, our mice were only exposed to ARVs *in utero*. Our findings suggest that ARV-associated neurotoxic effects experienced *in utero* can result in long-term consequences on brain morphology.

We observed a direct correlation between hippocampal CA1 volume and memory index in the ABC/3TC-exposed mice. Previous studies have reported an association between reduction in neuronal and astrocyte numbers in the hippocampus and impairments in hippocampal-dependent learning (Fitting et al., 2008; Akang, 2019); as encoding episodic memories requires the transmission of signals from granule cells of the dentate gyrus and CA1 pyramidal neurons in the hippocampus (Kitamura et al., 2015, 2017). However, the memory deficit observed in the TDF/FTC + ATV/r-exposed mice cannot be attributed to volumetric alterations in the brain, as we did not observe any volumetric differences in the brains of TDF/FTC + ATV/r-exposed mice compared to controls. Therefore, other pathways, such as neurotrophins and neurotransmitters could be involved. We observed downregulation of major glutamate receptors, AMPAR and NMDAR, in the hippocampus that may suggest an imbalance between inhibition/excitation in neural circuits that govern working memory and sociability in ART-exposed mice. Hyper-exploration also requires neurocircuitry involving the hippocampus (Van Praag et al., 1994), as well as the prefrontal cortex, and motor cortex (Johnson et al., 2012; Dong et al., 2021). Alteration in hippocampal mechanisms that govern oscillatory activity (rhythmic patterns of neural activity), which is regulated by AMPAR and NMDAR, can lead to altered exploratory activity and locomotion in rodents (Buzsáki, 2002; Rácz et al., 2009; Akgül and McBain, 2020). We also observed lower TrkB full-length levels in the hippocampus of TDF/FTC + ATV/r exposed mice, that could indicate that BDNF-dependent memory and locomotory pathways may be affected in these mice (Yamada and Nabeshima, 2004; Liu et al., 2015).

The focus of the current study was to understand the long-term deficits caused by *in utero* ART exposure on the development of the brain and related behavioral outcomes, as perinatal insults during critical periods of brain development can lead to long-lasting behavioral changes in adults and these changes may not be apparent until maturity (Black et al., 2017). Using a rodent model allowed us to focus on the impact of ART on the mechanisms of neurodevelopmental outcomes seen in CHEU without confounding factors such as HIV infection, and maternal, socioeconomic, and environmental factors (Wedderburn et al., 2019a; Bulterys et al., 2023). Our study identified neurophysiological deficits caused by *in utero* ART exposure in mice that can be correlated with the learning and cognitive delays, as well as attention deficits observed in CHEU (Burkey et al., 2015). However, our study has limitations. Our model does not include perinatal HIV exposure. While this is a limitation, as it does not directly mimic the CHEU exposures, it does allow us to study the specific effects of ARVs without the confounding of the virus effects on neurodevelopment. Additionally, CHEU often receive ARVs after birth as part of HIV prophylaxis, but our pups were not dosed postnatally. This likely also underestimates the ARV impact on brain development in our model, as some of the brain development that happens *in utero* for humans, happens postnatally in mice. Our expression analyses are limited to the hippocampus, and do not explore other brain regions that may also be implicated in the behavioral phenotypes we observed. Further, we only quantified mRNA levels. Additional studies looking at protein levels and localization would further our understanding.

In conclusion, we present evidence that perinatal exposure to ARVs is associated with long-term neurodevelopmental outcomes, including memory deficits, sex-dependent sociability deficits, increased exploration, and heightened locomotory activity. Such deficits may underlie some of the learning and motor difficulties observed in school aged CHEU. Further, we observed dramatic differences between the two different NRTI backbones tested, with brain volumetric reductions seen with ABC/3TC exposure, and hyperactivity seen with TDF/FTC exposure. Our findings underscore the importance of longitudinal follow-up of CHEU, inclusion of sex in all analyses, and more detailed evaluation of the effects of exposure to the different components of ART, with the goal of optimizing HIV treatment in pregnancy and health outcomes for CHEU.

## Data availability statement

The raw data supporting the conclusions of this article will be made available by the authors, without undue reservation.

## Ethics statement

The animal study was approved by University Health Network Animal Use Committee. The study was conducted in accordance with the local legislation and institutional requirements.

## Author contributions

SHD: Formal analysis, Investigation, Writing – original draft. KB: Formal analysis, Investigation, Writing – original draft. AS: Investigation, Writing – review & editing. SA: Investigation, Writing – review & editing. HTJM: Conceptualization, Writing – review & editing. LSC: Formal analysis, Investigation, Writing – review & editing. JGS: Conceptualization, Formal analysis, Writing – review & editing. LS: Conceptualization, Data curation, Formal analysis, Funding acquisition, Supervision, Writing – original draft.

## Footnotes

^1^ www.r-project.org

^2^ https://github.com/Mouse-Imaging-Centre/RMINC/
