# Supplementary material for: Perinatal exposure to atazanavir-based antiretroviral regimens in a mouse model leads to differential long-term motor and cognitive deficits dependent on the NRTI backbone
Source: Front Mol Neurosci. 2024 Apr 5;17:1376681. doi: 10.3389/fnmol.2024.1376681 (PMC11027900; doi:10.3389/fnmol.2024.1376681)
Supplement: Supplementary file 1 [file Data_Sheet_1.pdf]

Table S1: Summary of difference in means from mixed effects models for behavioural tests and expression analyses.

	**ABC/3TC + ATV/r**		**TDF/FTC + ATV/r**	
	**Male** Difference in mean (95% CI)	**Female** Difference in mean (95% CI)	**Male** Difference in mean (95% CI)	**Female** Difference in mean (95% CI)
**Open Field Maze**				
Total distance (m)	-1.6 (-7.2, 4.1)	-7.3 (-18.0, 3.3)	12.8 (2.5, 23)*	8.1 (0.2, 16.0)*
Mobile time (s)	-14.1 (-49.9, 21.6)	-47.3 (-111.8, 17.2)	66.5 (13.3, 119.7)*	41.4 (-3.7, 86.5)
Rearing (s)	-4.1 (-30.1, 22.0)	-19.0 (-50.0, 11.9)	80.6 (40.0, 121.3)***	70.2 (32.6, 107.8)***
Grooming (s)	16.6 (-3.3, 36.5)	32.6 (15.3, 49.8)***	-14.5 (-28.5, -0.44)*	-10.7 (-20.2, -1.3)*
Wall time (s)	-5.4 (-37.5, 26.6)	-8.4 (-25.9, 9.1)	6.7 (-10.2, 23.5)	11.4 (-1.8, 24.5)
Center time (s)	-2.8 (-25.3, 19.7)	5.3 (-13.8, 24.3)	-6.2 (-21.4, 8.9)	-2.9 (-17.6, 11.8)
**Light/Dark Box**				
Time in light box (s)	2.24 (-39.1, 43.6)	-1.77 (-24.3, 20.8)	20.8 (-9.7, 51.2)	33.0 (12.6, 53.5)**
**Zero Maze**				
Time in open area (s)	12.9 (0.8, 24.9)*	4.3 (-12.5, 21.1)	21.1 (11.6, 30.6)***	11.9 (-2.9, 26.7)
**Novel Object Recognition**	11.1 (-23.3, 45.6)	-13.5 (-73.0, 45.9)	55.7 (22.0, 89.4)**	86.7 (40.4, 133.0)***
Exploration time (s)	-15.5 (-22.4, -8.7)***	-13.4 (-26.0, -0.75)*	-2.2 (-9.6, 5.2)	10.8 (-1.8, 23.3)
Time with novel object (s)	-0.19 (-0.35, -0.025)*	-0.19 (-0.28, -0.11)***	-0.11 (-0.17, -0.046)**	-0.10 (-0.18, -0.027)**
Memory Index				
**Social Approach**				
Sociability Index	0.053 (-0.07, 0.18)	0.0057 (-0.09, 0.10)	-0.093 (-0.20, 0.017)	-0.10 (-0.18, -0.026)**
Social Novelty Index	0.037 (-0.09, 0.16)	-0.18 (-0.30, -0.05)**	0.020 (-0.12, 0.16)	-0.09 (-0.19, 0.012)
**RT-qPCR (AU)**				
BDNF	0.421 (0.045, 0.798)*	0.0016 (-0.16, 0.164)	0.090 (-0.12, 0.30)	0.0968 (-0.247, 0.44)
TrkB-full length receptor	-0.037 (-0.31, 0.24)	-0.07 (-0.32, 0.18)	-0.28 (-0.55, -0.009)*	-0.41 (-0.64, -0.17)**

TrkB-truncated receptor	0.063 (-0.028, 0.15)	0.18 (0.075, 0.29)**	-0.15 (-0.24, -0.054)**	0.063 (-0.04, 0.17)
PSD95	0.058 (-0.38, 0.50)	-0.26 (-0.48, -0.044)*	-0.053 (-0.519, 0.412)	-0.19 (-0.49, 0.10)
GluA1	-0.02 (-0.25, 0.21)	-0.085 (-0.22, 0.05)	-0.13 (-0.33, 0.07)	-0.17 (-0.29, -0.06)**
GluA2	-0.016 (-0.14, 0.11)	0.035 (-0.063, 0.13)	-0.23 (-0.33, -0.14)***	-0.030 (-0.13, 0.07)
GluN2A	0.083 (-0.25, 0.41)	-0.20 (-0.34, -0.058)**	0.11 (-0.17, 0.39)	-0.071 (-0.31, 0.17)
GluN2B	0.21 (-0.045, 0.46)	-0.24 (-0.42, -0.051)*	0.059 (-0.12, 0.24)	-0.10 (-0.32, 0.12)

Statistical comparisons stratified by sex, for all but MRI data, using mixed effects linear models with robust standard errors that included treatment as a categorical fixed effect, and litter as a random variable to account for litter effects. All comparison vs. control. *p<0.05, **p<0.01, ***p<0.001.

Table S2: Summary of difference in means from mixed effects models that include weight at postnatal day 3 for behavioural tests and MRI.

	ABC/3TC + ATV/r		TDF/FTC + ATV/r	
	**Male**<br>Difference in mean<br>(95% CI)	**Female**<br>Difference in mean<br>(95% CI)	**Male**<br>Difference in mean<br>(95% CI)	**Female**<br>Difference in mean<br>(95% CI)
**Open Field Maze**				
Total distance (m)	-2.4 (-8.5, 3.6)	-9.1 (-19.1, 0.97)	12.5 (2.2, 22.9)*	9.4 (1.3, 17.5)*
Mobile time (s)	-23.2 (-63.3, 16.9)	-62.9 (-128.1, 2.3)	65.2 (10.2, 120.2)*	47.3 (1.7, 92.9)*
Rearing (s)	1.1 (-27.0, 29.2)	-21.9 (-52.6, 8.8)	80.1 (39.5, 120.8)***	72.1 (35.6, 108.7)***
Grooming (s)	19.2 (-1.7, 40.2)	32.9 (14.8, 51.1)***	-12.8 (-26.6, 1.0)	-11.0 (-20.0, -2.0)*
Wall time (s)	1.0 (-33.8, 35.8)	-6.5 (-24.4, 11.3)	6.0 (-12.9, 24.9)	10.1 (-2.2, 22.3)
Center time (s)	-6.4 (-37.0, 24.3)	4.6 (-15.4, 24.6)	-4.0 (-22.3, 14.2)	-2.4 (-16.9, 12.1)
**Light/Dark Box**				
Time in light box (s)	3.8 (-37.6, 45.2)	-3.4 (-25.1, 18.2)	20.9 (-9.5, 51.2)	34.2 (12.7, 55.7)**
**Zero Maze**				
Time in open area (s)	10.8 (-4.3, 25.8)	2.0 (-14.7, 18.6)	20.3 (9.5, 31.1)***	12.2 (-3.5, 27.9)
**Novel Object Recognition**				
Exploration time (s)	-1.3 (-33.4, 30.9)	-16.8 (-74.8, 41.2)	48.4 (17.2, 79.6)**	88.0 (42.2, 133.9)***
Time with novel object (s)	-13.5 (-22.2, -4.9)**	-15.4 (-28.1, -2.7)*	-2.0 (-9.5, 5.4)	12.1 (-0.11, 24.3)
Memory Index	-0.18 (-0.35, -0.017)*	-0.19 (-0.27, -0.11)***	-0.11 (-0.17, -0.047)***	-0.11 (-0.19, -0.021)*
**Social Approach**				
Sociability Index	-0.002 (-0.11, 0.11)	0.024 (-0.071, 0.12)	-0.11 (-0.23, 0.007)	-0.10 (-0.18, -0.021)*
Social Novelty Index	0.021 (-0.11, 0.15)	-0.18 (-0.33, -0.035)*	0.013 (-0.12, 0.15)	-0.088 (-0.19, 0.019)

Statistical comparisons stratified by sex, for all but MRI data, using mixed effects linear models with robust standard errors that included treatment category and birth weight at postnatal day 3 as fixed effects, and litter as a random variable to account for litter effects. All comparison vs. control. *$p<0.05$, **$p<0.01$, ***$p<0.001$.

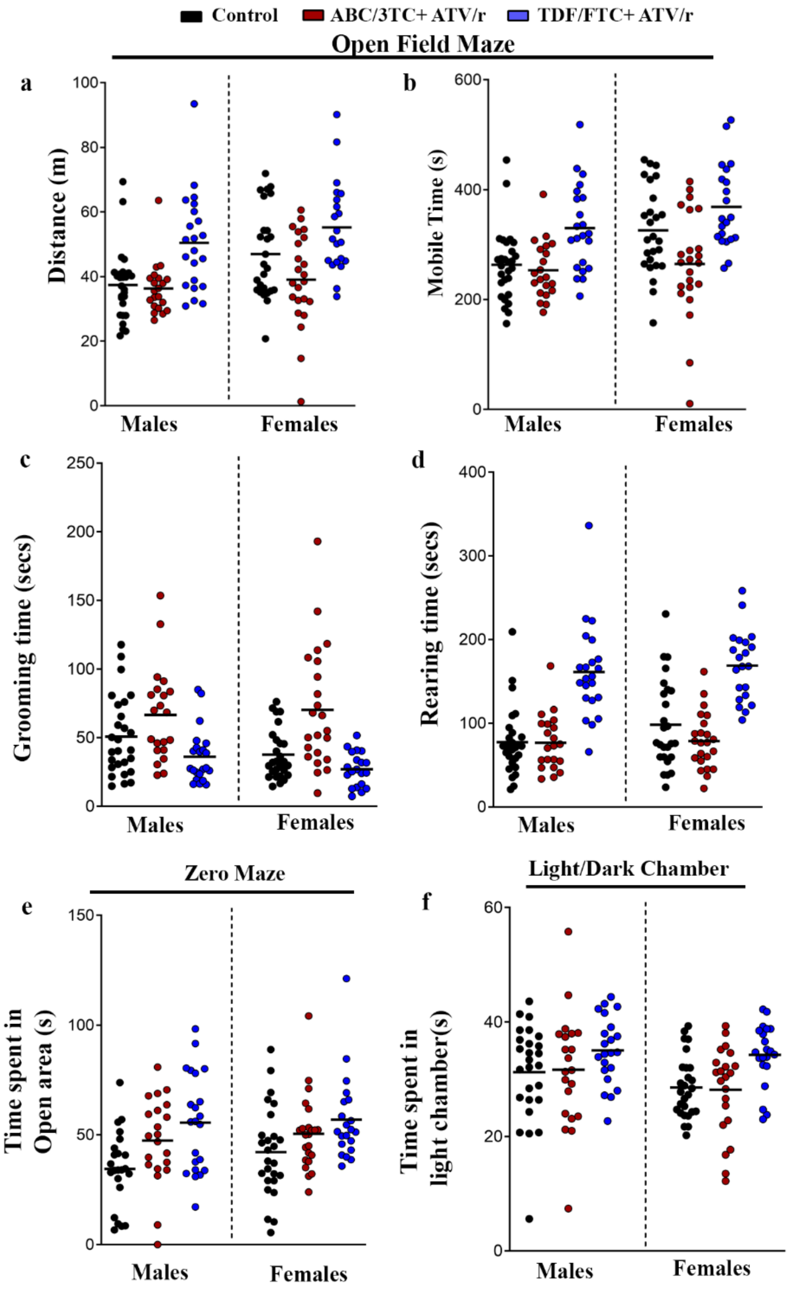

Figure S1: In utero exposure to TDF/FTC+ATV/r is associated with increased locomotor activity and exploratory behavior.

(A) distance travelled, **(B)** time mobile, **(C)** rearing time, and **(D)** grooming time in the open field test. **(G)** Time spent in the light box for the light/dark chamber test. **(H)** Time spent in the open area in the ero maze test. Data shown for male and female mice exposed to ABC/3TC+ATV/r (red dots) or TDF/FTC+ATV/r (blue dots). Each dot represents one animal. Lines indicate the median. For males: n=21 from 6 litters for ABC/3TC+ATV/r, n=22 from 6 litters for TDF/FTC+ATV/r, and n=28 from 9 litters for control. For females: n=27 from 8 litters for ABC/3TC+ATV/r, n=21 from 6 litters for TDF/FTC+ATV/r, n=27 from 9 litters for control. ABC, abacavir; 3TC, lamivudine; TDF, tenofovir; FTC, emtricitabine; ATV/r. ritonavir-boosted atazanavir. These are the raw data corresponding to Figure 2 in the main article. Please see Figure 2 for statistical comparisons.

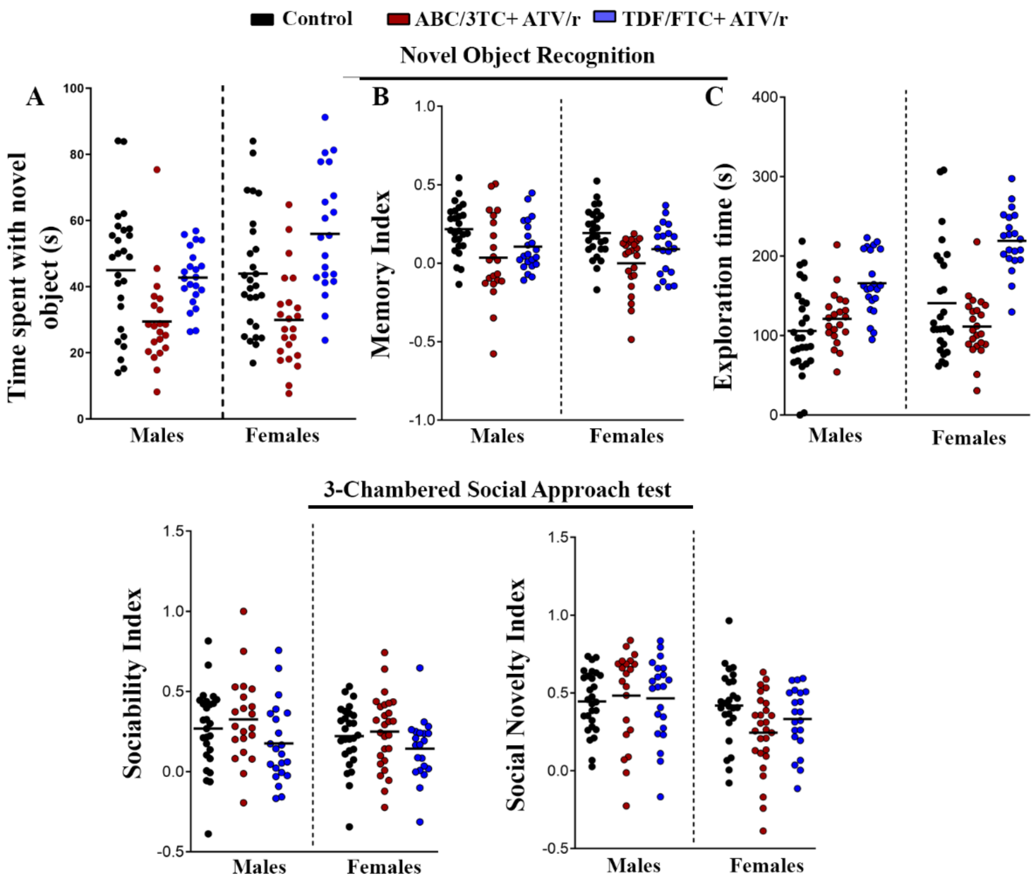

Figure S2: Working memory and sociability is impaired in mice exposed in utero to ART.

(A) Time with the novel object, **(B)** memory index, and **(C**) exploration time in the novel object recognition test. **(D)** Sociability index and **(E)** social novelty index in the 3-chamber social approach test. Data shown for male and female mice exposed to ABC/3TC+ATV/r (red dots) or TDF/FTC+ATV/r (blue dots). Each dot represents one animal. Lines indicate the median. For males: n=21 from 6 litters for ABC/3TC+ATV/r, n=22 from 6 litters for TDF/FTC+ATV/r, and n=28 from 9 litters for control. For females: n=27 from 8 litters for ABC/3TC+ATV/r, n=21 from 6 litters for TDF/FTC+ATV/r, n=27 from 9 litters for control. ABC, abacavir; 3TC, lamivudine; TDF, tenofovir; FTC, emtricitabine; ATV/r. ritonavir-boosted atazanavir. These are the raw data corresponding to Figure 3 in the main article. Please see Figure 3 for statistical comparisons.
